# Effect of Post-Weld Heat Treatment on Residual Stress and Fatigue Crack Propagation Behavior in Linear Friction Welded Ti-6Al-4V Alloy

**DOI:** 10.3390/ma18143285

**Published:** 2025-07-11

**Authors:** Sungkyoung Lee, Hyunsung Choi, Yunji Cho, Min Jae Baek, Hyeonil Park, Moo-Young Seok, Yong Nam Kwon, Namhyun Kang, Dong Jun Lee

**Affiliations:** 1 Aerospace Materials Center, Korea Institute of Materials Science, 797 Changwondaero, Changwon 51508, Republic of Korea; cutty0508@naver.com (S.L.); h.choi@kims.re.kr (H.C.); yun4666@kims.re.kr (Y.C.); qoralswo12@kims.re.kr (M.J.B.); hipark@kims.re.kr (H.P.); myseok@kims.re.kr (M.-Y.S.); kyn1740@kims.re.kr (Y.N.K.); 2 Department of Materials Science and Engineering, Pusan National University, Busan 46241, Republic of Korea

**Keywords:** linear friction welding, residual stress, fatigue crack propagation, post-weld heat treatment

## Abstract

In this study, the effects of post-weld heat treatment (PWHT) on residual stress distribution and fatigue crack propagation (FCP) behavior in linear friction welded (LFW) Ti-6Al-4V joints were investigated. Microstructural evolution in the weld center zone (WCZ), thermomechanically affected zone (TMAZ), heat-affected zone (HAZ), and base metal (BM) was characterized using scanning electron microscropy (SEM) and electron backscatter diffraction (EBSD). Mechanical properties were evaluated via Vickers hardness testing and digital image correlation (DIC)-based tensile testing. Residual stresses before and after PWHT were measured using the contour method. The LFW process introduced significant residual stresses, with tensile stresses up to 709.2 MPa in the WCZ, resulting in non-uniform fatigue crack growth behavior. PWHT at 650 °C and 750 °C effectively reduced these stresses. After PWHT, fatigue cracks propagated uniformly across the weld region, enabling reliable determination of crack growth rates. The average crack growth rates of the heat-treated specimens were comparable to those of the base metal, confirming that PWHT, particularly at 750 °C, stabilizes the fatigue crack path and relieves internal stress.

## 1. Introduction

Titanium and its alloys have long been valued for their exceptional combination of corrosion resistance, mechanical strength relative to weight, and resistance to fatigue and creep [1,2,3,4,5,6,7,8]. These attributes make them attractive candidates for use in high-performance fields such as aerospace, biomedical devices, and automotive engineering [9,10,11,12,13,14,15,16]. Nevertheless, the economic challenges of working with titanium, especially in aerospace manufacturing, remain significant. Conventional machining approaches typically remove a large portion of the starting material as scrap, often exceeding 90%, which drastically lowers material utilization and raises the buy-to-fly (BTF) ratio. Additionally, the intensive heat generated during machining shortens tool life and reduces dimensional control. To address these limitations, various research efforts have focused on alternative manufacturing processes that can enhance efficiency and minimize waste, thereby lowering production costs and improving the overall viability of titanium in structural applications.

One such strategy involves the use of linear friction welding (LFW) to fabricate aerospace components [17,18,19,20,21,22,23,24]. Traditionally, Ti-6Al-4V structural parts are machined from oversized ingots, forgings, or extrusions, resulting in significant material waste and high production costs due to inefficient buy-to-fly (BTF) ratios—sometimes as poor as 20:1. In contrast, LFW enables near-net-shape joining of smaller workpieces and eliminates the need for melting, thereby improving material utilization and minimizing typical fusion welding defects such as porosity and hot cracking [23,24]. These features make LFW an attractive technique for aerospace applications. However, despite these advantages, the broader industrial adoption of LFW remains limited, not only due to economic considerations, but also because of technical challenges such as high residual stresses in the weld region, microstructural heterogeneities across the weld interface, and unstable fatigue crack propagation behavior. These factors complicate the prediction of structural integrity and limit the qualification of LFWed components for safety-critical applications. Ongoing research is therefore needed to clarify the process–structure–property relationships and ensure consistent performance of LFW joints beyond niche applications such as blisk fabrication [21,22].

During linear friction welding (LFW) of Ti-6Al-4V alloys, the weld region typically consists of several distinct microstructural zones due to the localized thermal and mechanical conditions induced by the process: the weld center zone (WCZ), the thermomechanically affected zone (TMAZ), and the heat-affected zone (HAZ) [22]. The WCZ, situated at the weld interface, experiences the highest temperatures—often exceeding the β-transus—as well as the most intense plastic deformation. This results in dynamic recrystallization of the high-temperature β-phase and subsequent formation of a fine martensitic or Widmanstätten microstructure upon rapid cooling. The TMAZ is also plastically deformed but remains below the β-transus, leading to elongated and rotated α grains without full phase transformation. The HAZ is only thermally affected and does not undergo plastic deformation. These zones differ significantly in microstructure and mechanical response, and are therefore analyzed separately in studies of LFWed joints.

Previous studies have extensively investigated the microstructural evolution, hardness distribution, and residual stress profiles in Ti-6Al-4V linear friction welds (LFWed joints). These studies revealed that residual stresses, primarily tensile in nature and concentrated in the WCZ, play a critical role in determining structural integrity. Romero et al. [25], using synchrotron X-ray diffraction, confirmed that these stresses, generated by thermally induced strains during post-weld cooling and plastic deformation at elevated temperatures, often exceed 600 MPa. High residual stresses can significantly affect fatigue crack propagation (FCP) characteristics and compromise the accuracy of fatigue life predictions. To address this, researchers including Frankel et al. [26] have proposed post-weld heat treatment (PWHT) as a mitigation strategy. PWHT has been shown to significantly reduce residual stresses, in some cases by more than 90%. It also contributes to microstructural homogenization and mechanical property enhancement. However, despite these findings, a systematic understanding of how such microstructural and stress-state changes influence the direction, rate, and stability of fatigue crack growth remains limited. Detailed quantitative analyses on the interaction between residual stress distribution and fatigue crack behavior across the welded zones are still needed to optimize weld performance in demanding aerospace applications.

In addition to research on Ti-6Al-4V and other aerospace-grade alloys, recent studies have increasingly explored linear friction welding of dissimilar material combinations, such as α + β/β titanium alloys, aluminum–magnesium systems, and even aluminum–steel pairs [27,28,29,30,31,32]. These efforts aim to extend the applicability of LFW to broader engineering fields beyond aerospace, including automotive, marine, and energy industries. Such developments demonstrate the versatility and growing industrial relevance of LFW as a solid-state joining method for structurally critical applications.

This study was conducted to better understand the relationship between residual stress relaxation and fatigue crack propagation behavior in LFWed Ti-6Al-4V under varying PWHT conditions for stress relief (SR), through quantitative assessments using the contour method, and fatigue crack growth tests. Specifically, microstructural changes were characterized using Electron Backscatter Diffraction (EBSD), and hardness measurements were performed to evaluate local mechanical property variations across the weld zones. Tensile properties were evaluated through uniaxial testing, during which Digital Image Correlation (DIC) was employed to monitor strain localization and deformation behavior across the weld. Additionally, fatigue crack propagation behavior was examined to clarify how residual stress relaxation influences crack growth direction, rate, and stability. These combined approaches provide a comprehensive understanding of how thermal and mechanical changes induced by PWHT affect the integrity of LFWed titanium structures.

## 2. Materials and Methods

**Figure 1:**
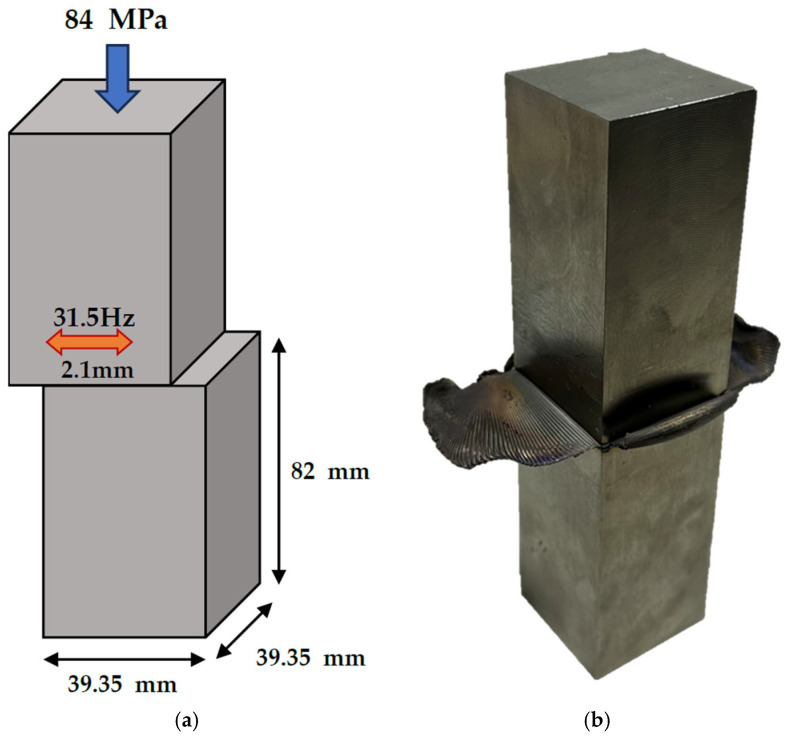
(**a**) Schematic diagram of the linear friction welding (LFW) setup and specimen geometry, and (**b**) linear friction welded specimen.

To investigate the effect of residual stress and microstructure on fatigue crack behavior in Ti-6Al-4V welded by LFW, rolled Ti-6Al-4V (AMS4911, Grade 5) alloy plates (TIMET, Wentzville, MO, USA) were used. The chemical composition shown in Table 1 was provided by the manufacturer based on a mill certificate for the as-received Ti-6Al-4V alloy. The LFW specimens were machined as blocks measuring 39.5 mm × 39.5 mm × 82 mm, as illustrated in Figure 1. The following LFW process parameters were set: welding pressure 84 MPa, forge time 5.2 s, frequency 31.5 Hz, and amplitude 2.1 mm. The initial total length of the two workpieces was 164 mm, which was reduced to approximately 160 mm after welding, indicating a total burn-off length of about 4 mm (2 mm from each side). To evaluate the effect of post-weld heat treatment (PWHT) on residual stress relief and microstructural evolution, the welded specimens were heat-treated at 650 °C and 750 °C, respectively. The specimens were heated to the target temperature at a rate of 5 °C/min, held for 30 min, and then furnace-cooled to room temperature.

The temperatures of 650 °C and 750 °C were selected based on both industrial guidelines and previous research findings. The 650 °C condition corresponds to the upper end of the typical stress relief range (540–650 °C) recommended by alloy suppliers such as ATI for Ti-6Al-4V. In addition, several previous studies have employed heat treatments at 750 °C and 800 °C to investigate their effects on microstructure and fatigue behavior in linear friction welded Ti-6Al-4V and related alloys [31,32,33]. Accordingly, 650 °C was chosen to represent a conventional stress relief condition, while 750 °C was included to allow comparison with literature-reported treatments and to assess the influence of increased thermal exposure on residual stress and fatigue crack propagation.

**Table 1:** Chemical composition of Ti-6Al-4V alloy used in this study (mass%) Adapted from Ref. [34].

Elements	C	Fe	Al	V	N	O	H	Ti
mass (%)	0.009	0.18	6.25	4.26	0.003	0.09	0.0025	Bal.

**Figure 2:**
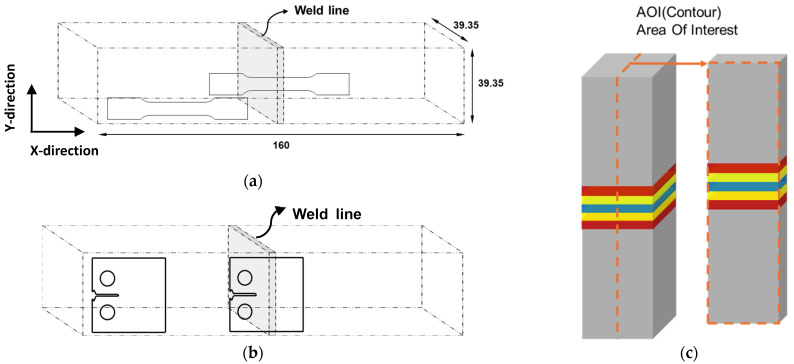
Locations of (**a**) tensile test specimens, (**b**) compact tension (CT) specimens used for fatigue crack propagation test, and (**c**) the cutting surface for contour method.

Tensile specimens were machined from both the welded and the base metal (BM) regions (Figure 2a), following the ASTM E8 sub-size specification. The specimens had a gauge length of 24 mm, a width of 6 mm, and a thickness of 1.5 mm. Three specimens were tested for each region to evaluate repeatability and allow statistical comparison. The tensile tests were performed on a universal testing machine (UT-100E, MTDI, Deajeon, Republic of Korea) following ASTM E8 standards, at a crosshead speed of 1.5 mm/min. Local strain distribution during the tests was analyzed using Digital Image Correlation (DIC, ARAMIS ver.6.3.1). 

Hardness testing was conducted using a Mitutoyo HM-200 Micro Vickers hardness tester (Mitutoyo, Kawasaki, Japan) in accordance with ASTM E384. A test load of 500 gf was applied for 15 s (HV0.5 condition). The surface of the LFW welds was polished up to 2000 grit to ensure uniformity. Measurements were performed on cross-sectioned specimens, with indentations made at 0.1 mm intervals from the weld center line outward to both sides, covering up to 8 mm from the center in each direction—from the WCZ through the TMAZ and the HAZ, into the BM.

For microstructural characterization of the BM, the HAZ, the TMAZ, and the WCZ, the surfaces were mechanically polished up to 4000 grit and then final-polished with oxide polishing solution (OP-S) suspension (Struers, Copenhagen, Denmark). Microstructural observation and EBSD analysis were conducted using a Hitachi SU6600 SEM (Hitachi, Tohyo, Japan). Average grain size was determined using OIM Analysis software ver. 8.6 based on EBSD data, following the procedures outlined in ASTM E2627. Grain boundaries were defined using a minimum misorientation angle of 15°, and the reported values represent number-weighted averages. The reported grain size represents a number-weighted average, and primarily corresponds to α-phase grains.

The internal residual stress of linear friction welds was measured using the contour method. This technique involves cutting the specimen using a wire electrical discharge machining (Wire-EDM) process, measuring the deformation profile at the cut surface, and applying the inverse displacement as a boundary condition in a finite element model to back-calculate the internal stress. In this study, the specimen was sectioned perpendicular to the loading direction (Y-direction) using a Wire-EDM machine at a feed rate of 0.5 mm/min, with a wire diameter of 0.25 mm. Surface height profiles of the cut surfaces were measured using the SPSM-10 system (FusionENG Co., Ltd., Suwon, Republic of Korea), which operates based on the principles of a 3D coordinate measuring machine. The system is equipped with a confocal laser sensor (CL-L015G, Keyence, Osaka, Japan), offering a vertical resolution of 0.003 μm. The acquired displacement data were smoothed and used as boundary conditions in a finite element analysis conducted using ABAQUS ver.2024 (Dassault Systèmes, Vélizy-Villacoublay, France) to calculate the residual stress field in the Y-direction.

To compare the crack growth rates at the weld interface under constant stress intensity factor (ΔK), crack propagation was conducted across the joint interface and compared with that of the base metal. Fatigue crack propagation (FCP) tests were conducted in accordance with ASTM E647 using compact tension (CT) specimens with a width (W) of 25.4 mm and thickness (B) of 6.35 mm (Figure 2b). Both the BM and the WCZ specimens were polished with 2000-grit paper to minimize surface defects and oxidation prior to testing. Tests were conducted using an MTS Landmark 100 kN hydraulic fatigue testing system, equipped with Crack Opening Displacement (COD) gauges (MTS systems, Eden Prairie, MN, USA). to measure crack length during testing. A pre-crack of 6.5 mm was introduced at a stress ratio (R) of 0.1 and 20 Hz frequency, using the constant ΔK method with ΔK = 12 MPa√m. The main test followed the same loading conditions, allowing the crack to grow up to 18 mm to ensure it propagated through the weld interface. FCP tests measure crack growth rate under a constant load amplitude to evaluate how ΔK affects material behavior as the crack progresses. In this study, the test was designed to observe fatigue crack propagation behavior across the LFW joint.

## 3. Results and Discussion

### 3.1. Microstructure and Properties After LFW

**Figure 3:**
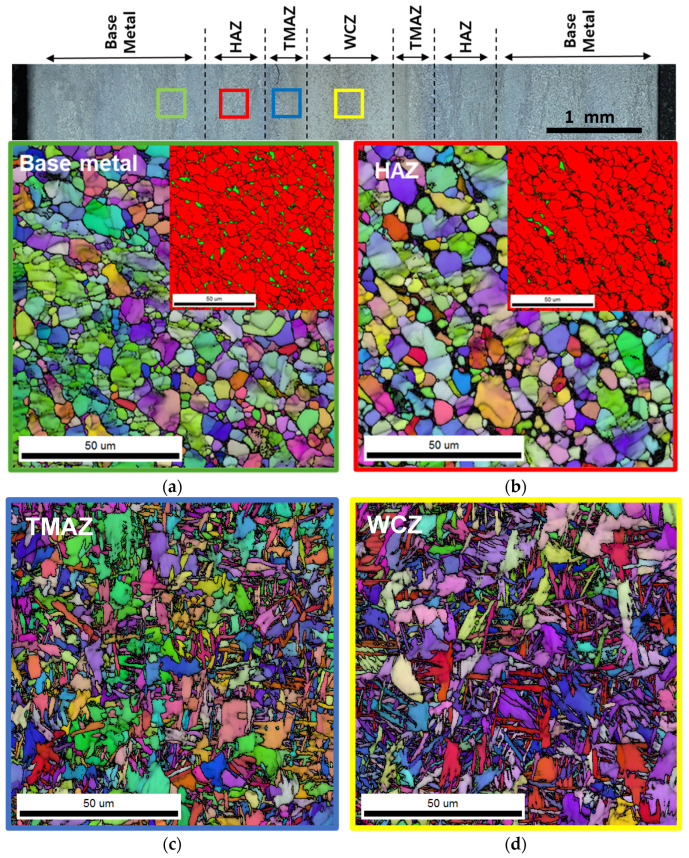
EBSD microstructures of (**a**) the BM, (**b**) the HAZ, (**c**) the TMAZ, and (**d**) the WCZ regions after LFW. Phase maps are additionally shown in the upper right corner of (**a**,**b**); red and green represent α and β phases, respectively. Phase maps were not included for (**c**,**d**) because the β phase appears in a fine acicular form in these regions, making it difficult to resolve using EBSD.

The microstructure of the LFWed specimen, including phase distribution and grain morphology, was characterized by EBSD, as shown in Figure 3. The BM exhibits a bimodal α + β phase structure, with the α phase having an average grain size of approximately 3.4 μm, as confirmed by the phase map in Figure 3a. In the HAZ, a similar bimodal α + β microstructure is observed, with the α-phase grains being slightly coarser (~4.0 μm) as a result of thermal exposure during welding. The TMAZ shows a refined microstructure with acicular α grains (~2.3 μm), formed through thermomechanical deformation and dynamic recrystallization near the sub-β-transus temperature, followed by cooling to room temperature. In the WCZ, bonding occurs at temperatures above the β-transus under high thermal and mechanical loads, and subsequent rapid cooling results in the formation of a fine α′ martensitic structure.

**Figure 4:**
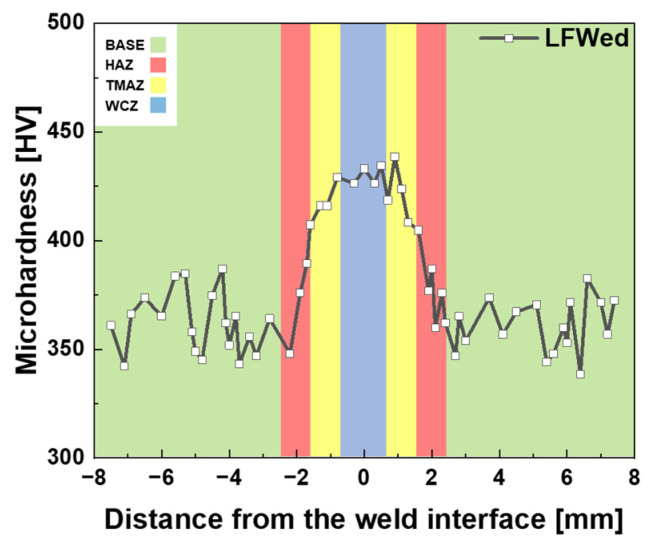
Microhardness distribution across the LFW joint from BM to WCZ.

Hardness measurements revealed that the BM and the HAZ retained values between 350 and 380 HV, suggesting limited thermal impact on the HAZ during welding. These results are shown in Figure 4. The TMAZ showed increased hardness values of 380–420 HV, attributed to dynamic recrystallization and the formation of acicular α grains. The WCZ exhibited the highest hardness (410–440 HV), which can be explained by the formation of fine α′ martensite [31,32,33]. This phase formed as a result of bonding in the β-phase region under high temperature and pressure during LFW, followed by rapid cooling, as also observed in the EBSD analysis (Figure 3d). These results highlight a gradient in mechanical properties across the weld, governed by the local thermal and deformation histories.

**Figure 5:**
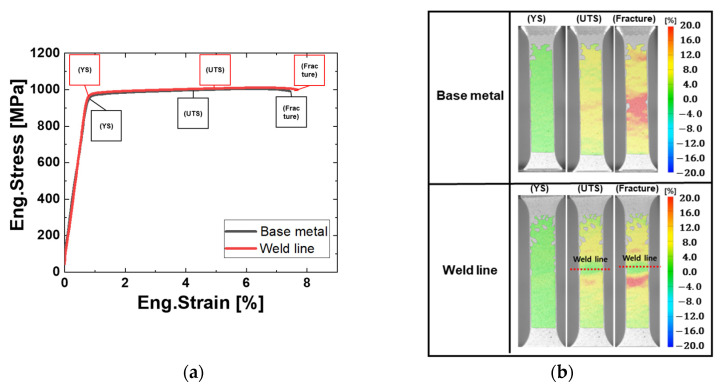
(**a**) Engineering stress–strain curves obtained from tensile tests of the BM and welded specimen containing the weld line at the center; (**b**) DIC results showing the effective strain distribution for each specimen during tensile testing.

The tensile properties of the BM and the welded specimen containing the weld line at the center were comparable, with results summarized in Figure 5. The BM exhibited a yield strength (YS) of 981.0 ± 5.3 MPa, a ultimate tensile strength (UTS) of 1017.5 ± 2.0 Mpa, and elongation at fracture of 7.7 ± 0.3%. After LFW, the welded specimen—including the weld line—showed comparable tensile properties, with a YS of 968.1 ± 7.3 Mpa, a UTS of 1001.9 ± 5.3 Mpa, and elongation at fracture of 7.9 ± 0.4%, indicating negligible mechanical degradation. DIC analysis during tensile testing revealed uniform strain distribution up to UTS in the BM specimen, followed by localized necking and fracture at the center. In contrast, the welded specimen exhibited localized post-yield deformation primarily in the HAZ and the TMAZ, with minimal plastic strain observed in the WCZ. This behavior is consistent with the micro-Vickers hardness results, where the WCZ—containing high-strength α′ martensite formed during LFW—resisted plastic deformation. Consequently, strain was concentrated in the thermally softened HAZ, leading to eventual failure in that region.

### 3.2. Effects of Post-Weld Heat Treatment

**Figure 6:**
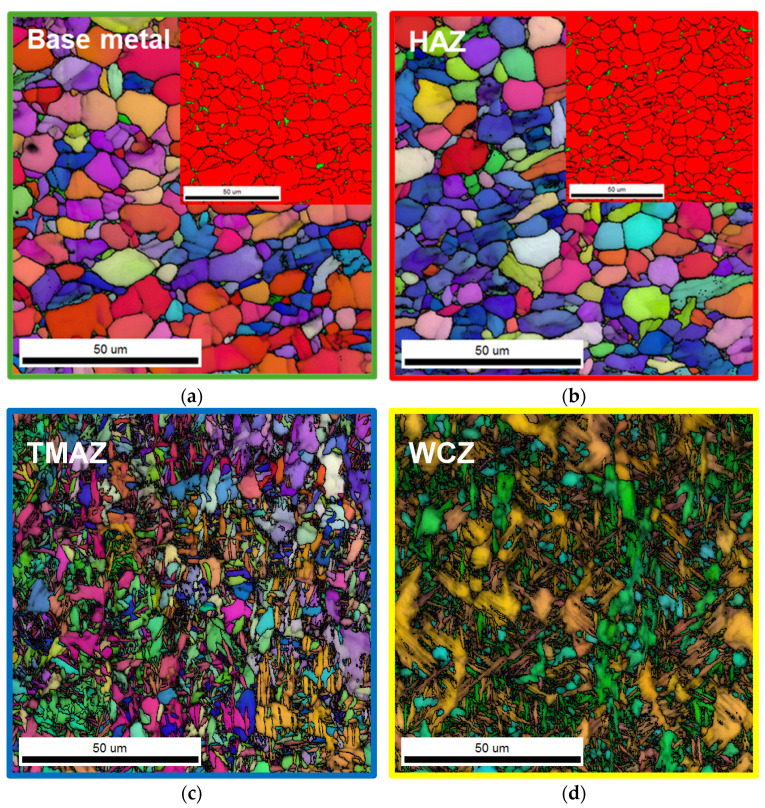
EBSD microstructures of (**a**) the BM, (**b**) the HAZ, (**c**) the TMAZ, and (**d**) the WCZ regions after post-weld heat treatment at 650 °C. Phase maps are additionally shown in the upper right corner of (**a**,**b**); red and green represent α and β phases, respectively. Phase maps were not included for (**c**,**d**) because the β phase appears in a fine acicular form in these regions, making it difficult to resolve using EBSD.

**Figure 7:**
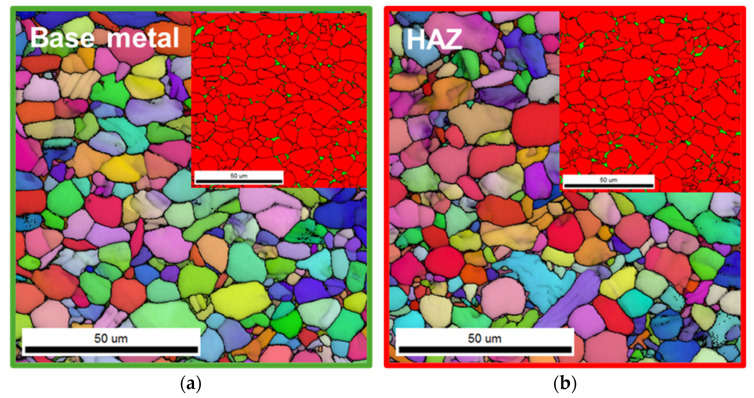

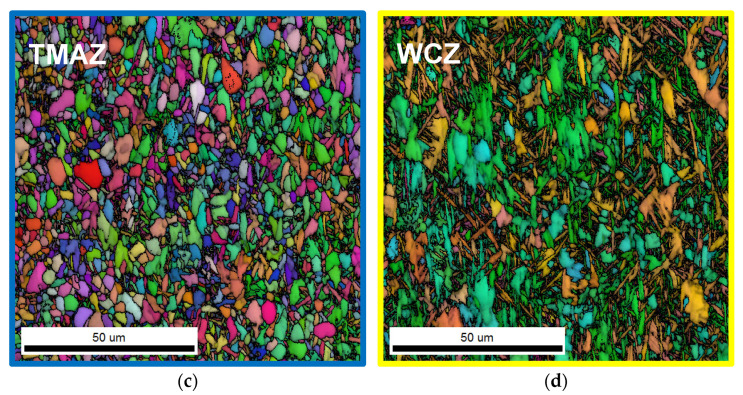
EBSD microstructures of (**a**) the BM, (**b**) the HAZ, (**c**) the TMAZ, and (**d**) the WCZ regions after post-weld heat treatment at 750 °C. Phase maps are additionally shown in the upper right corner of (**a**,**b**); red and green represent α and β phases, respectively. Phase maps were not included for (**c**,**d**) because the β phase appears in a fine acicular form in these regions, making it difficult to resolve using EBSD.

Microstructural evolution after post-weld heat treatment revealed grain coarsening and morphological recovery in the weld zones, especially at 750 °C. Figure 6 and Figure 7 present representative EBSD maps supporting these observations. Similar to the as-welded condition, the BM and the HAZ retained a bimodal α + β phase structure, while the TMAZ exhibited fine acicular α grains, and the WCZ displayed a Widmanstätten structure. Following heat treatment, the average grain size of α phase in the BM increased from 3.4 μm to 4.5 μm and 4.7 μm after treatment at 650 °C and 750 °C, respectively (Table 2). However, the grain sizes of α phase in the HAZ, the TMAZ, and the WCZ remained largely unchanged. These results indicate that while thermal exposure during stress relief promoted grain growth in the BM, the microstructure in the weld-affected regions was stable and not significantly altered by heat treatment at either temperature. It should be noted that direct comparison of grain size values across different weld zones (e.g., BM, TMAZ, WCZ) is not meaningful due to differences in their morphological characteristics (e.g., equiaxed vs. acicular or martensitic structures). Grain size analysis in this study was performed to assess relative changes within each zone before and after PWHT.

**Figure 8:**
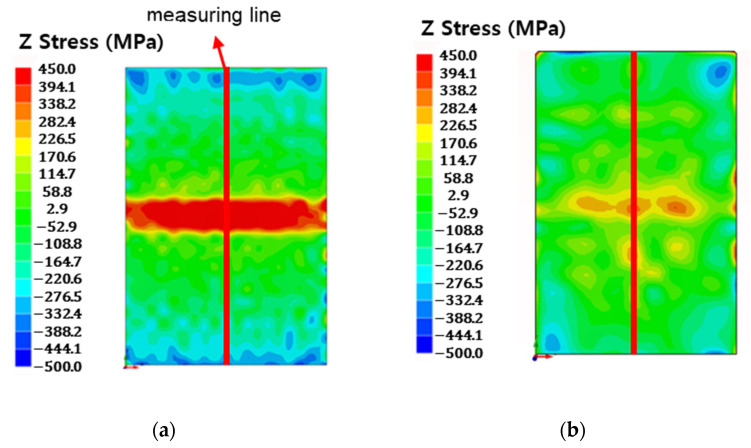

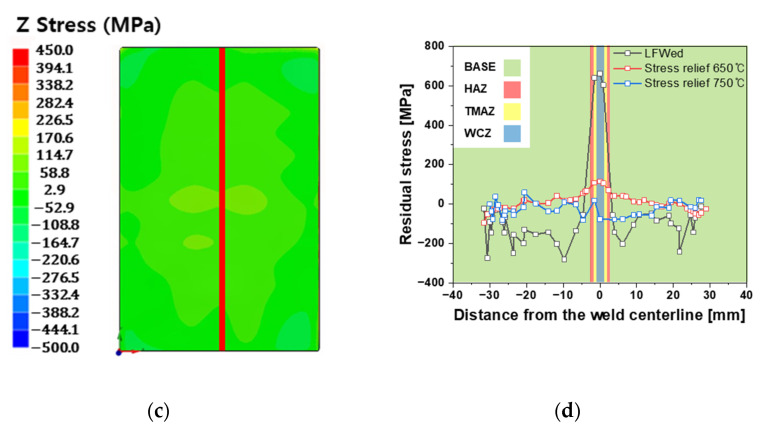
Residual stress mapping using the contour method (**a**) before and (**b**,**c**) after post-weld heat treatment at 650 °C and 750 °C, respectively. (**d**) Plot of residual stress distributions at the center line before and after post-weld heat treatment.

Residual stress distributions before and after post-weld heat treatment were measured using the contour method. A comparison of the results is presented in Figure 8. In the as-welded condition, the WCZ exhibited a peak tensile residual stress of 709.2 MPa, while compressive residual stresses up to −310.3 MPa were observed in the adjacent TMAZ, HAZ, and BM regions. After stress relief heat treatment at 650 °C, residual stresses in the weld-affected zones and the BM were largely relieved, whereas the WCZ still retained a reduced tensile stress of 114.4 MPa. Following 750 °C heat treatment, residual stress throughout the entire weld, including the WCZ, was significantly reduced, indicating that higher temperature annealing was more effective in alleviating internal stresses.

Residual stress measurements using the contour method revealed that tensile residual stresses were concentrated in the WCZ after the LFW process, while compressive residual stresses were present in the surrounding regions. After stress relief heat treatments, the magnitudes of both tensile and compressive residual stresses decreased. Notably, heat treatment at 750 °C resulted in more complete stress relaxation compared to that at 650 °C.

**Figure 9:**
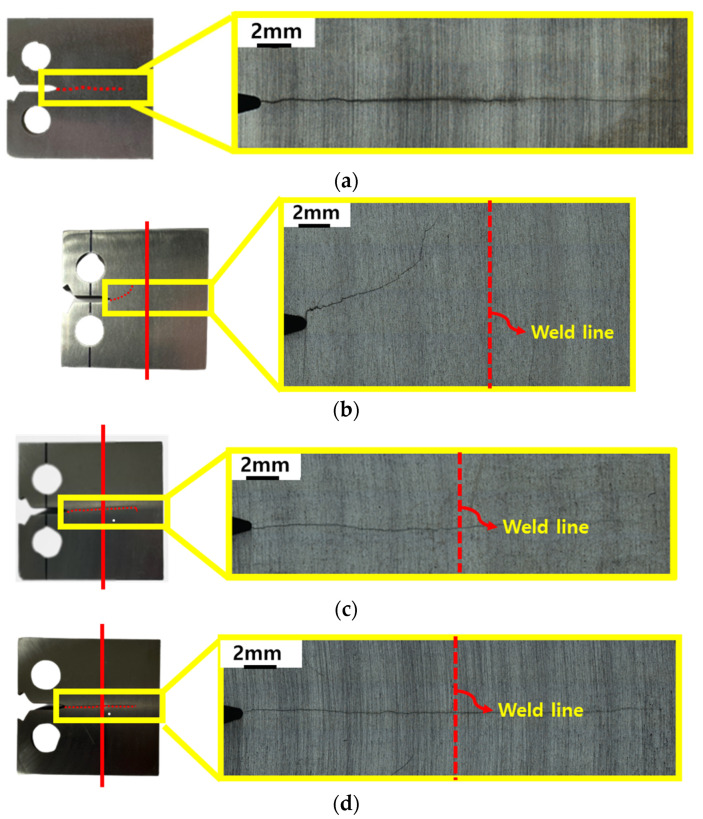
Fatigue crack paths observed in (**a**) the BM, (**b**) as-welded, (**c**) 650 °C heat-treated, and (**d**) 750 °C heat-treated specimens.

**Figure 10:**
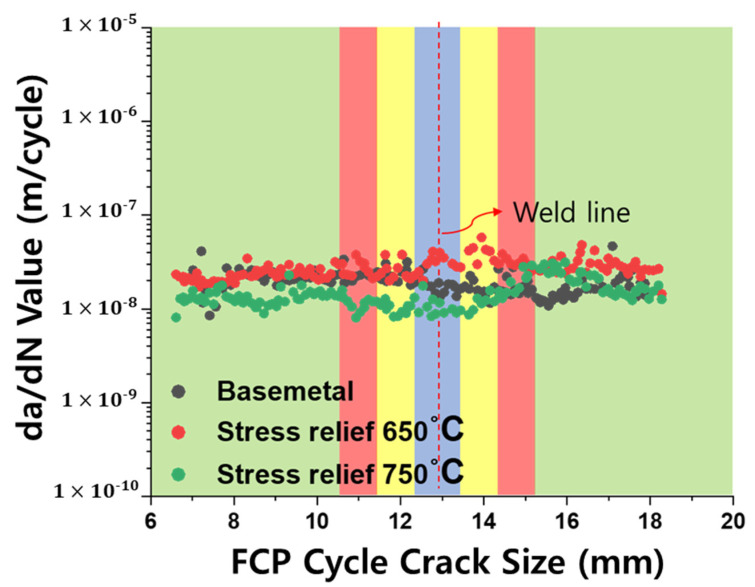
Crack growth rate (da/dN) versus crack length for the BM and LFWed specimens under ΔK = 12 MPa√m.

Fatigue crack propagation (FCP) tests were conducted to evaluate the effect of post-weld heat treatment on crack growth behavior. Post-test specimens and the corresponding crack growth rates as a function of crack position are shown in Figure 9 and Figure 10, respectively. For the BM, fatigue cracks propagated linearly and uniformly under a constant ΔK of 12 MPa√m, with an average growth rate of 1.96 × 10^−8^ m/cycle (Figure 9a). In contrast, in the as-welded LFW specimen—where the crack front was oriented perpendicular (90°) to the weld interface—the fatigue crack failed to propagate through the WCZ, instead deviating from its original path (Figure 9b). Due to this deviation, the crack did not grow in a straight, measurable path, and thus, the crack growth rate could not be reliably calculated for this condition. However, in the specimens subjected to post-weld heat treatment at 650 °C and 750 °C, crack propagation occurred uniformly across the weld zone, similar to that of the BM (Figure 9c,d). The average fatigue crack growth rates were 2.74 × 10^−8^ m/cycle and 1.48 × 10^−8^ m/cycle, respectively, as summarized in Table 3. According to a paper that studied the fatigue crack growth rate of Ti-6Al-4V alloy [35], a rate of approximately 10^−8^ m/cycle was observed at ΔK = 12 MPa√m, which is similar to the conditions in this study. This can be considered a similar value to the fatigue crack growth rate obtained in this test.

In the as-welded condition, the deflection of the fatigue crack path in the LFWed specimen indicates that the crack could not penetrate the WCZ. This phenomenon is believed to be closely associated with the residual stresses within the weld, particularly the shear stress component. According to the Maximum Principal Stress Theory, a crack propagates along a plane perpendicular to the direction of maximum principal stress. The directions of the principal stress and crack growth are defined respectively as

(1) $$\sigma_{max}=\frac{\sigma_{xx}+\sigma_{yy}}{2}\pm \sqrt{{\left(\frac{\sigma_{xx}-\sigma_{yy}}{2}\right)}^{2}+\tau_{xy}^{2}}$$

(2) $$\tan2\theta_{p}=\frac{2\tau_{xy}}{\sigma_{xx}-\sigma_{yy}}$$

where $\sigma_{xx}$ and $\sigma_{yy}$ are the normal stresses in the x and y directions, respectively, and $\tau_{xy}$ is the shear stress. As shown in Equation (2), the crack propagation direction is highly influenced by the shear stress component. In uniaxial and biaxial tensile conditions where only normal stress components exist, cracks tend to propagate perpendicular to the loading direction, which corresponds to the direction of maximum principal stress. In the context of fatigue crack propagation, cracks typically grow perpendicular to the direction of the applied tensile load at the crack tip. Thus, the deviation observed in the as-welded specimen is indicative of non-uniform stress distribution and the presence of significant shear stress near the weld, which alters the direction of crack growth. According to strain mapping studies [36] conducted on Ti alloys before and after PWHT, residual shear strain components were observed not only in the weld zone but also in the surrounding regions. These strains were identified in both the α and β phases, with the β phase often exhibiting relatively higher levels of residual strain. In addition, complex distributions of axial residual strains were detected throughout the welded area. These findings indicate that a multiaxial residual strain field comprising both shear and axial components exists within and around the weld. After PWHT, a significant reduction in these strain components was observed. This suggests that residual shear and axial strains can influence the direction of fatigue crack propagation by preventing cracks from growing strictly perpendicular to the applied loading direction.

In addition to residual stress and strain effects, the influence of material flow during the LFW process may also be a contributing factor to crack path deviation. As summarized in previous literature [22], the TMAZ in Ti-6Al-4V linear friction welds is often characterized by elongated and reoriented α grains aligned along the direction of oscillation. This flow-like structure results from severe plastic deformation below the β-transus temperature and can introduce microstructural anisotropy, which may affect crack propagation stability. This mechanism represents an additional possible contributor to crack deflection in as-welded specimens. Furthermore, the high hardness observed in the WCZ of the as-welded specimen suggests increased local crack resistance. According to previous findings [35], fatigue cracks tend to propagate more slowly in β-annealed-like microstructures compared to conventional α + β structures. Since the WCZ exhibits such a microstructure, it may have contributed to the observed deviation by resisting crack advance through the weld center. This interpretation is further supported by recent work on welded steel structures, which demonstrated that residual stress fields can significantly alter crack tip driving forces and must be considered alongside microstructure when evaluating crack propagation behavior [37].

Following post-weld stress relief heat treatment, especially at 750 °C, the surrounding compressive residual stresses were significantly reduced. Consequently, fatigue cracks propagated in a linear path across the weld, similar to the base metal. In the specimen heat-treated at 650 °C, however, a slight increase in crack growth rate was observed in the WCZ. This is likely attributable to remaining tensile residual stresses that were not fully relieved. The results measured by contour (Figure 8b) show that the residual stress in the WCZ is not completely relieved. Since the residual stress measured by contour is stress in the Y direction, it is a different direction from the X direction, where force is applied during the fatigue crack propagation test. However, in the paper [25] that studied various directions, it can be seen that tensile residual stresses mostly exist in the X, Y, and Z directions in the WCZ after the LFW process. Therefore, there is a possibility that the residual stress in the X direction, the same as the fatigue crack propagation load, after the 650 °C heat treatment is not completely relieved, and tensile residual stresses remain. Because these stresses act in the same direction as the external load, they raise the effective stress intensity at the crack tip, accelerating crack opening and growth.

Conversely, the specimen treated at 750 °C exhibited a marginally reduced average crack growth rate. This reduction is attributed to grain coarsening in the base metal during heat treatment. Fatigue cracks typically propagate along grain boundaries; thus, larger grains reduce the number of boundary intersections and potential crack initiation sites, ultimately slowing down crack propagation. In addition, the coarsened grain boundaries may exhibit higher resistance to crack advance, further contributing to reduced growth rates. Moreover, other microstructural recovery mechanisms, such as dislocation density reduction or partial transformation of martensite to α phase, may also have contributed to the improved crack growth resistance.

Although the WCZ exhibited a β-annealed-like microstructure—previously shown to suppress fatigue crack growth in Ti-6Al-4V [35]—this study did not observe a significantly slower growth rate in the WCZ compared to the base metal. This may be due to the relatively high ΔK value (12 MPa√m) used in the experiment, where the dominant influence of residual stress outweighs the microstructural effects. At lower ΔK levels, microstructural differences are more pronounced, but this influence diminishes as ΔK increases. Additionally, in the 650 °C condition, the presence of residual tensile stress in the WCZ likely counteracted the crack growth retardation normally expected from the martensitic microstructure.

The LFW process offers advantages over conventional fusion welding by avoiding filler material and associated defects such as solidification porosity or excessive grain coarsening. However, the process still induces rapid thermal cycling, leading to non-negligible residual stresses. These stresses can unpredictably affect fatigue life and crack growth characteristics.

This study confirmed that significant residual stresses in the as-welded condition can hinder stable crack propagation, resulting in path deviation and irregular growth behavior. These instabilities may challenge the structural reliability of LFWed components in service. However, after appropriate post-weld heat treatment, cracks were observed to propagate along paths perpendicular to the applied load, and the fatigue behavior of the weld region became comparable to that of the base metal. These results clearly demonstrate the effectiveness of PWHT—particularly at 750 °C—in stabilizing fatigue crack growth through residual stress relaxation.

While the present findings demonstrate the beneficial effects of PWHT on fatigue crack behavior in linear friction welded Ti-6Al-4V, the study was conducted under constant-amplitude loading at a single ΔK level and in ambient air conditions. These controlled parameters do not fully capture the complexity of real-world service environments, where variable loading, elevated temperatures, or corrosive media may alter fatigue performance. Future work incorporating such factors would be valuable to further validate the applicability of the results to practical aerospace components.

## 4. Conclusions

Post-weld residual stress measurements using the contour method confirmed the presence of tensile residual stresses concentrated in the weld center zone (WCZ) and compressive residual stresses in the adjacent regions following linear friction welding (LFW). These residual stresses were significantly reduced after post-weld heat treatment (PWHT), with more effective stress relaxation observed at 750 °C compared to 650 °C. 

Fatigue crack propagation (FCP) tests demonstrated that, in the as-welded condition, cracks could not penetrate the WCZ and instead deviated, likely due to the influence of internal residual stresses, particularly shear components. This crack deflection was suppressed in specimens subjected to PWHT, where cracks propagated uniformly across the weld zone, similar to the behavior in the base metal. The average crack growth rates for the PWHT specimens at 650 °C and 750 °C were 2.74 × 10^−8^ m/cycle and 1.48 × 10^−8^ m/cycle, respectively, confirming that higher heat treatment temperature promoted better stress relaxation and enhanced fatigue resistance.

Despite the slight increase in crack growth rate in the WCZ after 650 °C heat treatment due to residual tensile stress, PWHT at 750 °C effectively minimized this issue. The study highlights the critical role of residual stress management in improving the fatigue performance of Ti-6Al-4V LFW joints, emphasizing the necessity of optimized heat treatment protocols for structural integrity in high-performance applications.

## Figures and Tables

**Table 2:** Average grain size of α phase in (**a**) the BM, (**b**) the HAZ, (**c**) the TMAZ, and (**d**) the WCZ regions before and after post-weld heat treatment.

Specimens	BM	HAZ	TMAZ *	WCZ *
**LFWed**	3.4 ± 2.8	4.0 ± 3.2	2.3 ± 1.6	2.3 ± 1.8
**SR650**	4.5 ± 3.6	4.4 ± 3.2	2.2 ± 1.5	2.1 ± 1.7
**SR750**	4.7 ± 3.3	4.6 ± 3.5	2.4 ± 1.4	2.0 ± 1.5

* The grain size values for TMAZ and WCZ should be interpreted with caution, as these regions exhibit acicular or martensitic microstructures, for which conventional average grain size measurement is not directly applicable.

**Table 3:** Average fatigue crack growth rates of the BM and LFWed specimens under ΔK = 12 MPa√m.

Specimens	Average Crack Growth Rate (m/cycle, × 10^−8^)	Standard Deviation (m/cycle, × 10^−8^)
Base Metal	1.96	0.55
SR650	2.74	0.63
SR750	1.48	0.51

## Data Availability

The original contributions presented in this study are included in the article. Further inquiries can be directed to the corresponding authors.
